# Supplementary material for: Metabolomic Profiling of the Immune Stimulatory Effect of Eicosenoids on PMA-Differentiated THP-1 Cells
Source: Vaccines (Basel). 2019 Oct 9;7(4):142. doi: 10.3390/vaccines7040142 (PMC6963534; doi:10.3390/vaccines7040142)
Supplement: Supplementary file 1 [file vaccines-07-00142-s001.pdf]

Supplementary Materials:

TNF-α production

Table S1: Effect of eicosenoid compounds on the production of TNF-α cytokine in the presence and absence of LPS on PMA-differentiated THP-1 cells (n =3).

	TNF-α concentration (pg/ml)									
	Sample Only					Sample + LPS				
**Eicosenoid compounds**	n = 1	n = 2	n = 3	Mean	RSD	n = 1	n = 2	n = 3	Mean	RSD
**(11E)-OH**	422	375	273	356.67	21.36	592	586	595	591.00	0.78
**(11E)-ester**	52	29	4	28.33	84.73	537	578	581	565.33	4.35
**(11E)-acid**	182	155	37	124.67	61.85	599	615	604	606.00	1.35
**Media**	100	83	98	93.67	9.92					
**LPS**	572	572	587	577.00	1.50					

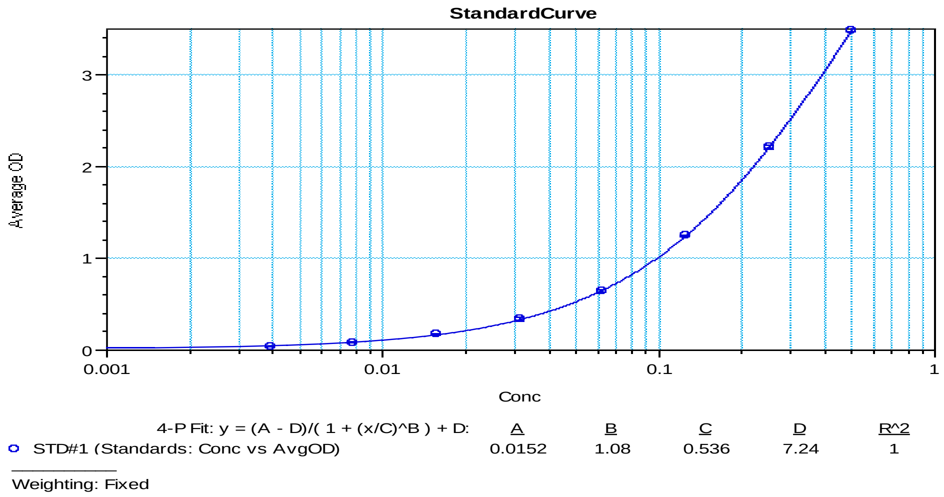

Figure S1: A representative 4-parameter logistic plot of TNF-α standard samples of 8 points showing the values of A, B, C, and D constants and the calibration equation with a perfect fit (R^2 = 1.0). The data represents the mean ± SD of optical density (OD) values for duplicate standard concentrations (n = 2).

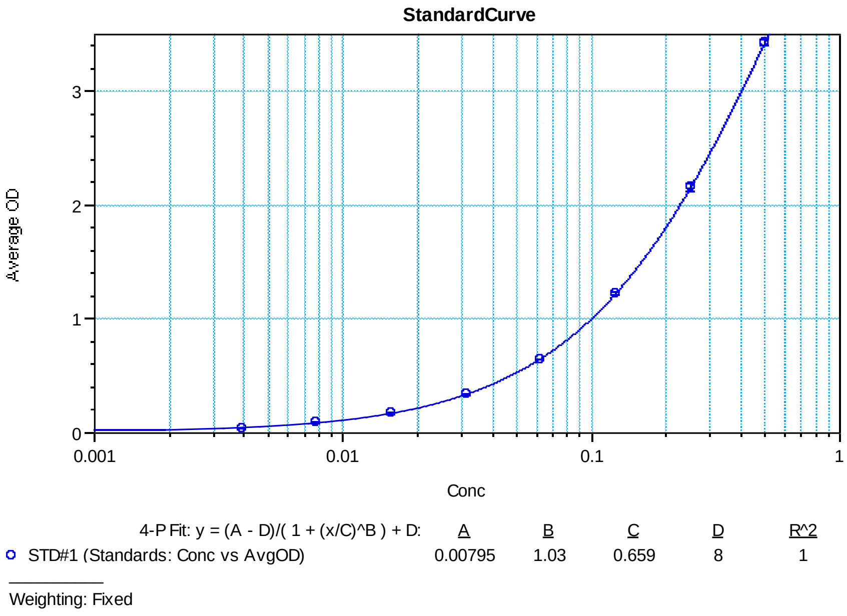

Figure S2: A representative 4-parameter logistic plot of TNF-α standard samples of 8 points showing the values of A, B, C, and D constants and the calibration equation with a perfect fit ($R^2 = 1.0$). The data represents the mean ± SD of optical density (OD) values for duplicate standard concentrations ($n = 2$).

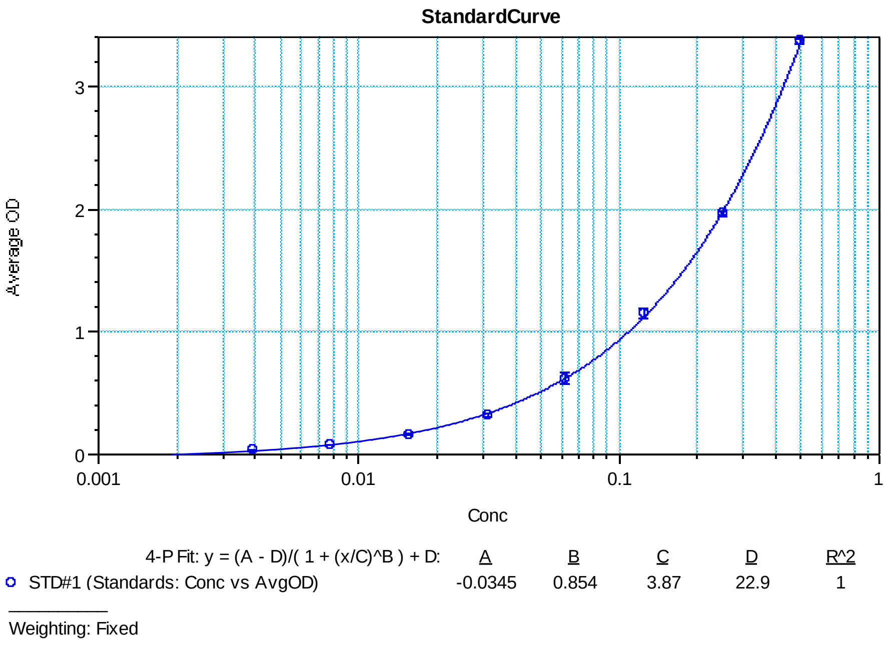

Figure S3: A representative 4-parameter logistic plot of TNF-α standard samples of 8 points showing the values of A, B, C, and D constants and the calibration equation with a perfect fit ($R^2 = 1.0$). The data represents the mean ± SD of optical density (OD) values for duplicate standard concentrations ($n = 2$).

IL-1β Production

Table S2: Effect of eicosenoid compounds on the production of IL-1β cytokine in the presence and absence of LPS on PMA-differentiated THP-1 cells (*n* = 3).

	IL-1β concentration (pg/ml)									
	**Sample Only**					**Sample + LPS**				
**Eicosenoid compounds**	*n* = 1	*n* = 2	*n* = 3	Mean	RSD	*n* = 1	*n* = 2	*n* = 3	Mean	RSD
**(11E)-OH**	90	86	26	67.33	53.24	158	110	85	117.67	31.53
**(11E)-ester**	73	22	10	35.00	95.58	152	129	135	138.67	8.60
**(11E)-acid**	124	83	35	80.67	55.22	181	123	120	141.33	24.33
**Media**	47	47	37	43.67	13.22					
**LPS**	82	78	68	76	9.48					

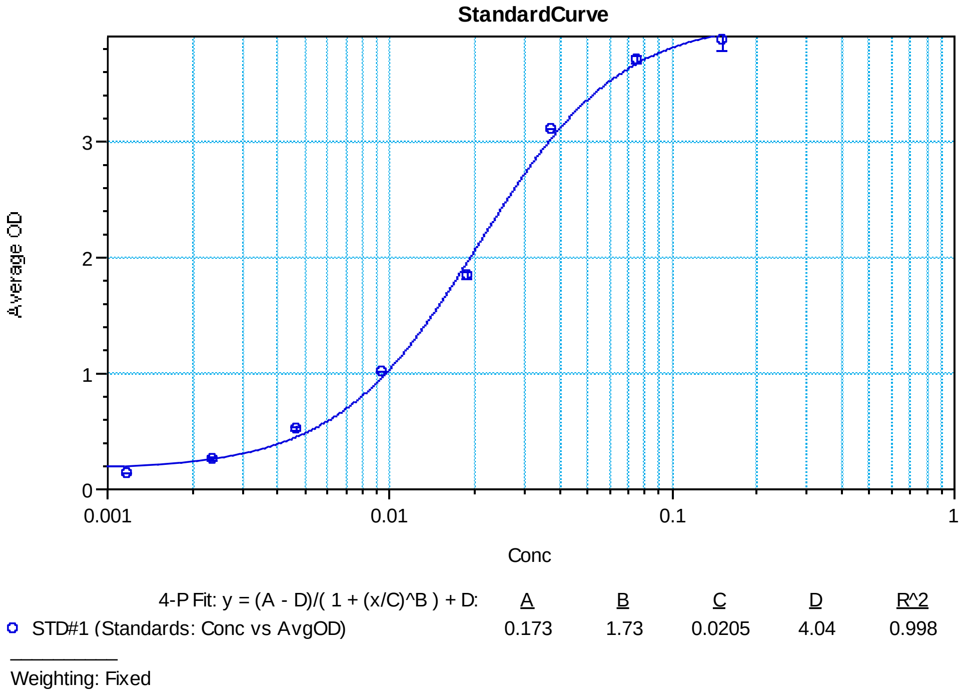

Figure S4: A representative 4-parameter logistic plot of IL-1β standard samples of 8 points showing the values of A, B, C, and D constants and the calibration equation with a perfect fit ($R^2 = 0.998$). The data represents the mean ± SD of optical density (OD) values for duplicate standard concentrations (*n* = 2).

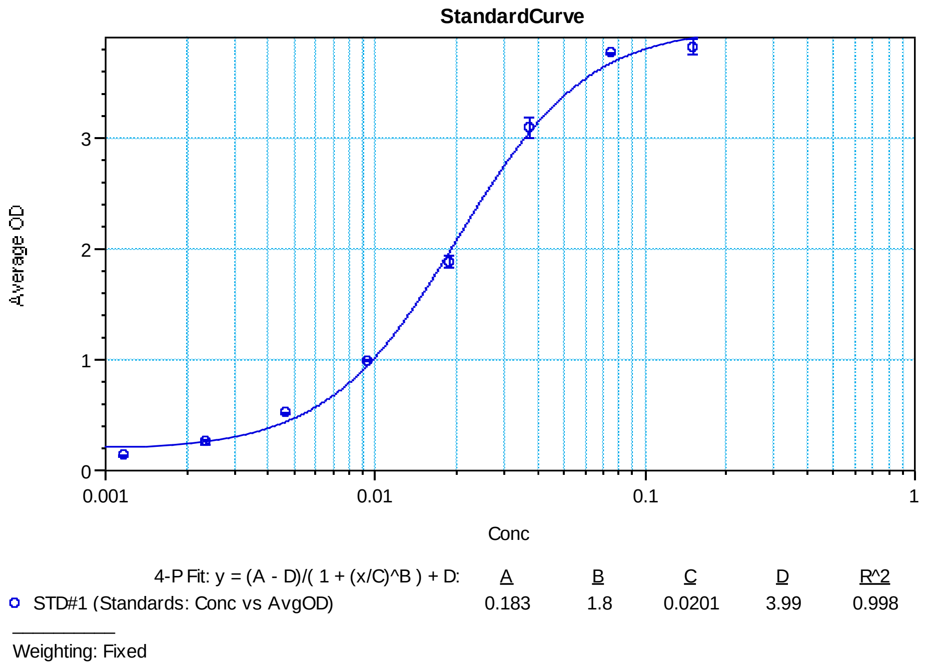

Figure S5: A representative 4-parameter logistic plot of IL-1β standard samples of 8 points showing the values of A, B, C, and D constants and the calibration equation with a perfect fit ($R^2 = 0.998$). The data represents the mean ± SD of optical density (OD) values for duplicate standard concentrations ($n = 2$).

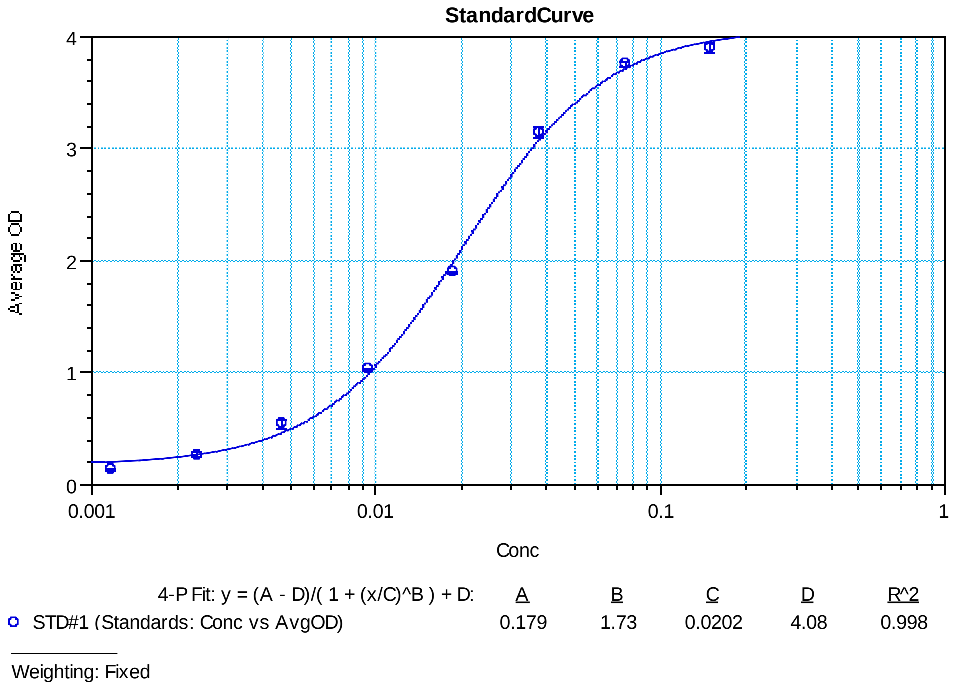

Figure S6: A representative 4-parameter logistic plot of IL-1β standard samples of 8 points showing the values of A, B, C, and D constants and the calibration equation with a perfect fit ($R^2 = 0.998$). The data represents the mean ± SD of optical density (OD) values for duplicate standard concentrations ($n = 2$).

IL-6 Production

Table S3: Effect of eicosenoid compounds on the production of IL-6 cytokine in the presence and absence of LPS on PMA-differentiated THP-1 cells (n = 3).

	**IL-6 *concentration (pg/ml)***									
	**Sample only**					**Sample + LPS**				
**Eicosenoid compounds**	n = 1	n = 2	n = 3	Mean	RSD	n = 1	n = 2	n = 3	Mean	RSD
**(11E)-OH**	<2.0	<2.0	<2.0	n/a	n/a	38	62	71	57	29.93
**(11E)-ester**	<2.0	<2.0	<2.0	n/a	n/a	<2.0	<2.0	<2.0	<2.0	n/a
**(11E)-acid**	<2.0	<2.0	<2.0	n/a	n/a	21.00	47.00	39.00	35.67	37.34
**Media**	<2.0	<2.0	<2.0	n/a	n/a					
**LPS**	88.00	104.00	114.00	102.00	12.86					

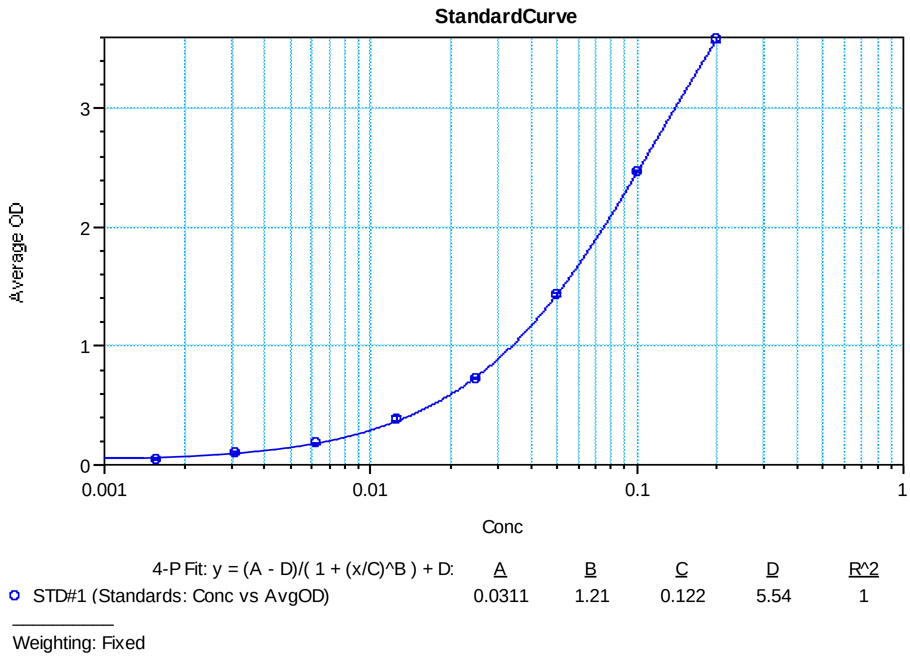

Figure S7: A representative 4-parameter logistic plot of IL-6 standard samples of 8 points showing the values of A, B, C, and D constants and the calibration equation with a perfect fit ($R^2 = 1$). The data represents the mean ± SD of optical density (OD) values for duplicate standard concentrations ($n = 2$).

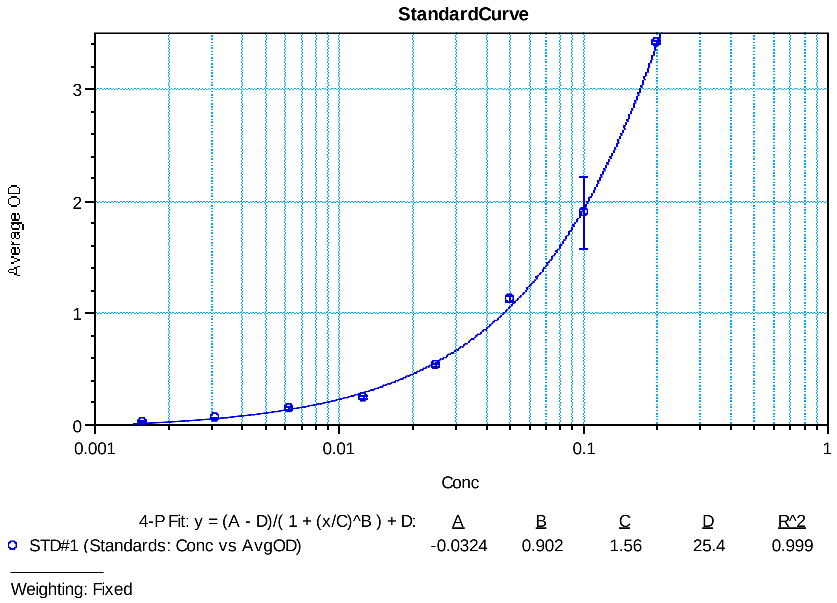

Figure S8: A representative 4-parameter logistic plot of IL-6 standard samples of 8 points showing the values of A, B, C, and D constants and the calibration equation with a perfect fit ($R^2 = 0.999$). The data represents the mean ± SD of optical density (OD) values for duplicate standard concentrations ($n = 2$).

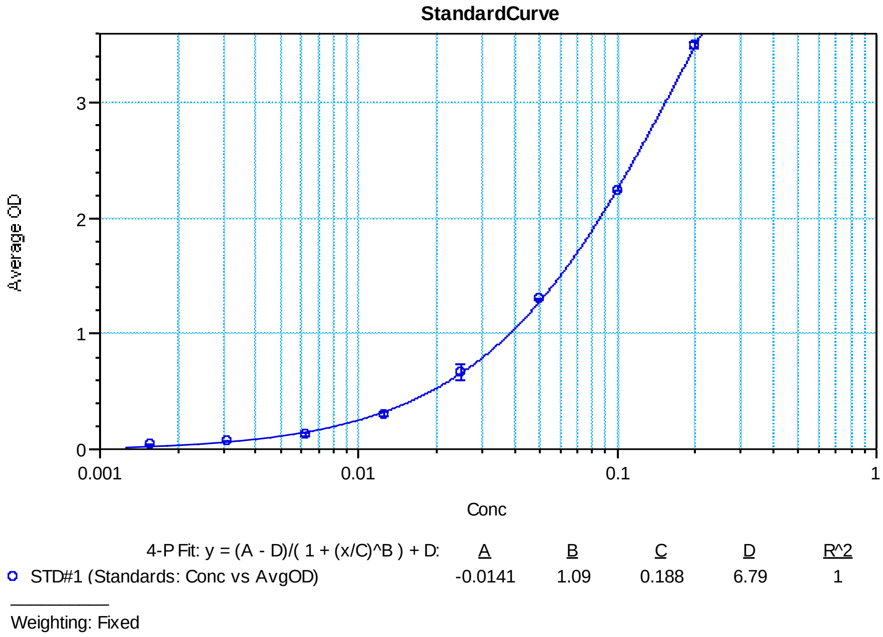

Figure S9: A representative 4-parameter logistic plot of IL-6 standard samples of 8 points showing the values of A, B, C, and D constants and the calibration equation with a perfect fit ($R^2 = 1$). The data represents the mean ± SD of optical density (OD) values for duplicate standard concentrations ($n = 2$).

IL-10 Production

Table S4: Effect of eicosenoid compounds on the production of IL-10 cytokine in the presence and absence of LPS on PMA-differentiated THP-1 cells ($n = 3$).

	**IL-10** ***concentration (pg/ml)***									
	**Sample Only**					**Sample + LPS**				
**Eicosenoid compound s**	$n = 1$	$n = 2$	$n = 3$	Mean	RSD	$n = 1$	$n = 2$	$n = 3$	Mean	RSD
**(11E)-OH**	10.00	10.00	6.00	8.67	26.65	14.00	15.00	17.00	15.33	9.96
**(11E)-ester**	3.00	<2.0	<2.0	3	n/a	<2.0	3.00	6.00	4.5	47.14
**(11E)-acid**	5.00	4.00	5.00	4.67	12.37	8.00	12.00	12.00	10.67	21.65
**Media**	5.00	2.00	9.00	5.33	65.85					
**LPS**	20.00	21.00	23.00	21.33	7.16					

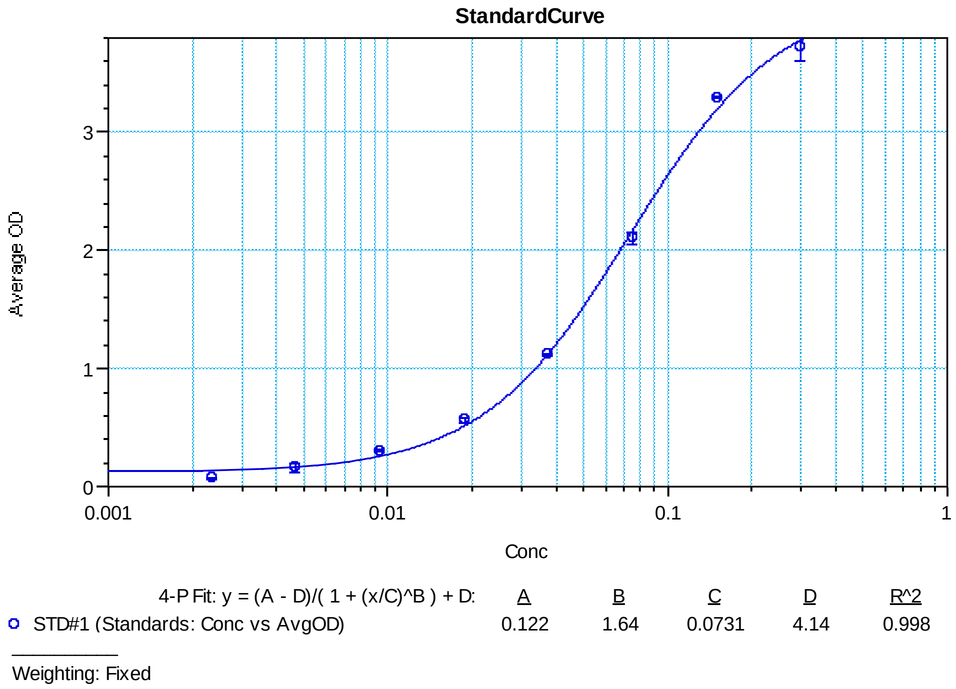

Figure S10: A representative 4-parameter logistic plot of IL-10 standard samples of 8 points showing the values of A, B, C, and D constants and the calibration equation with a perfect fit (R^2 = 0.998). The data represents the mean ± SD of optical density (OD) values for duplicate standard concentrations (n = 2).

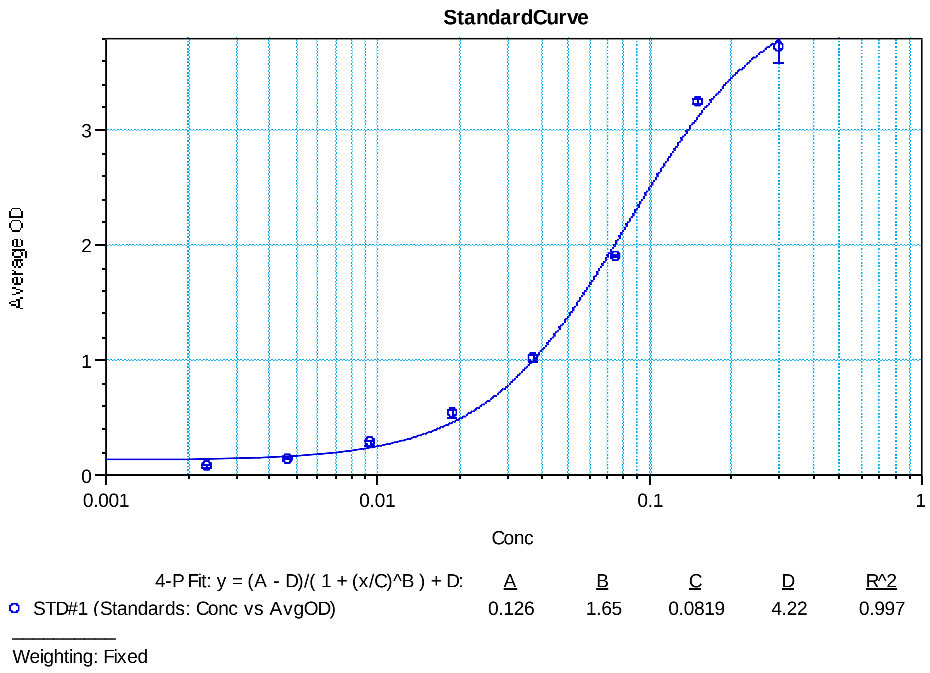

Figure S11: A representative 4-parameter logistic plot of IL-10 standard samples of 8 points showing the values of A, B, C, and D constants and the calibration equation with a perfect fit (R^2= 0.997). The data represents the mean ± SD of optical density (OD) values for duplicate standard concentrations (n = 2).

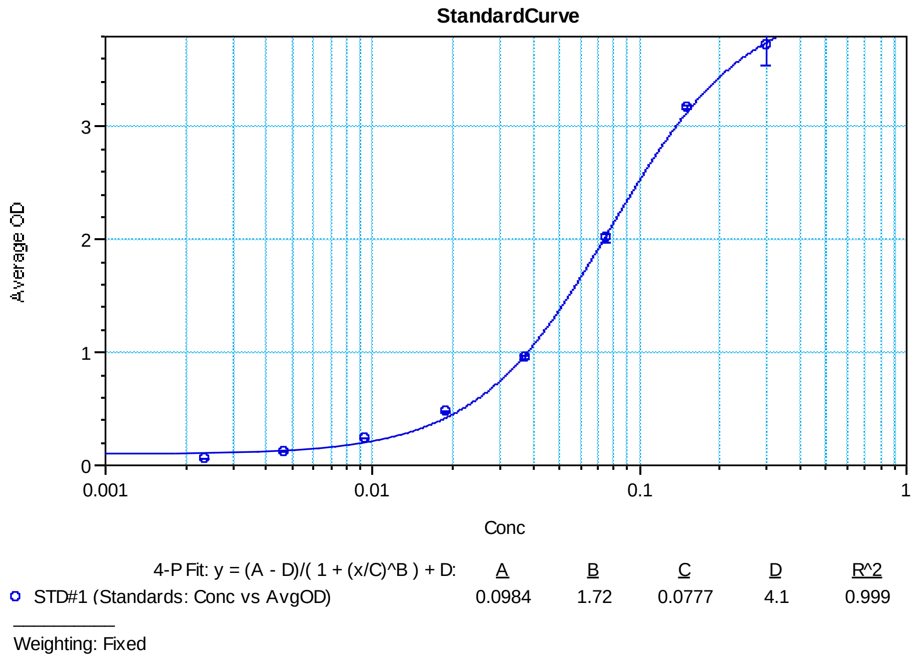

Figure S12: A representative 4-parameter logistic plot of IL-10 standard samples of 8 points showing the values of A, B, C, and D constants and the calibration equation with a perfect fit (R^2= 0.999). The data represents the mean ± SD of optical density (OD) values for duplicate standard concentrations (n = 2).

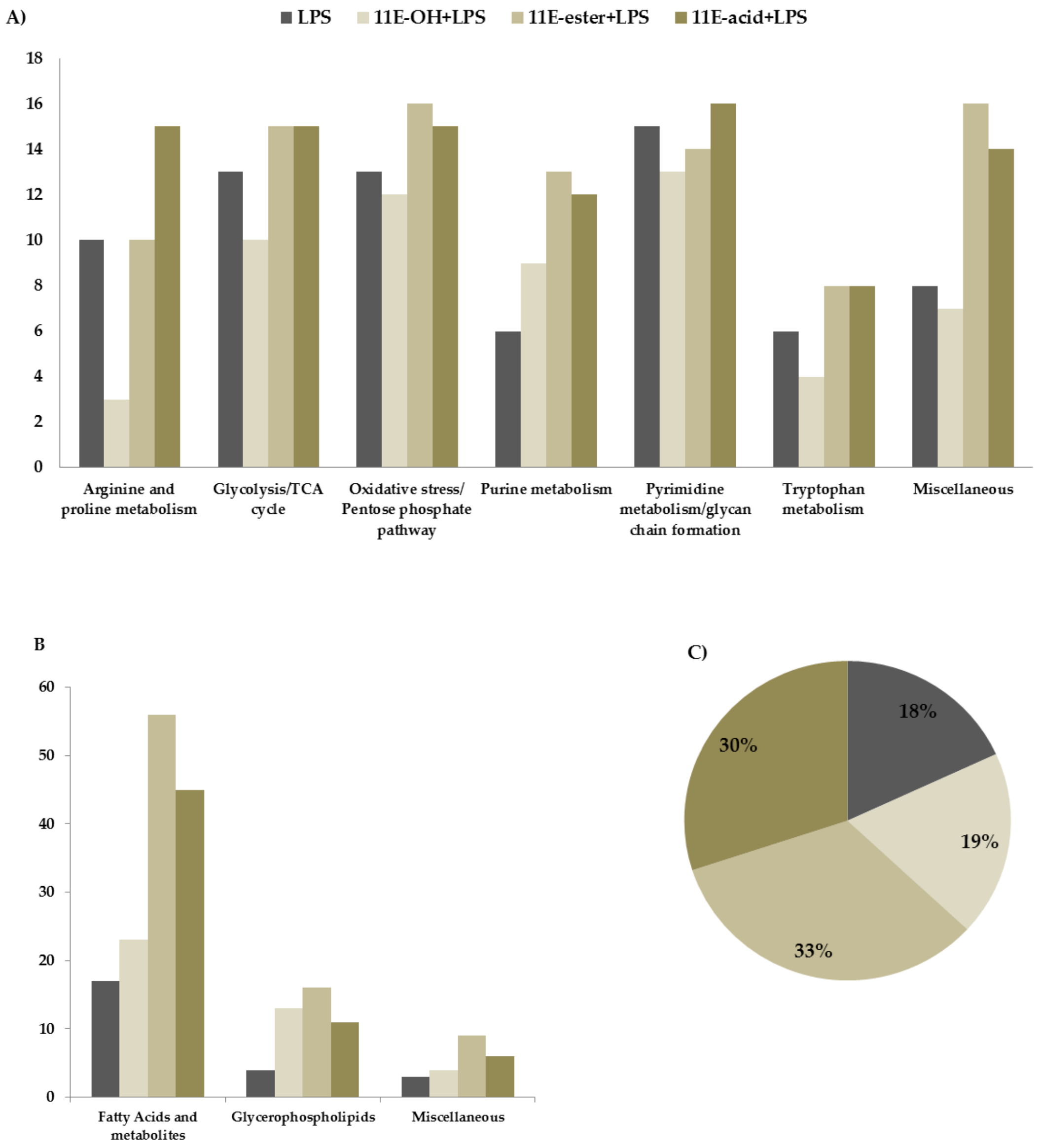

Figure S13: Changes in metabolites by treatment with LPS alone or in combination with one of three forms of eicosenoid derivatives when compared with untreated control cells. (**A**) Metabolites separated on a ZIC-pHILIC column and (**B**) on an ACE C4 column. (**C**) Percentage of significantly changed metabolites by treatments. (LPS: Lipopolysaccharide; (11E-OH): (Z)-11-eicosenol; (11E-ester): Eicosenoate; (11E-acid): Eicosenoic acid).

Table S5. Additional changed non-polar metabolites in THP-1 cells treated with lipopolysaccharide (LPS), alone or in combination with one of three synthetic forms of honey bee eicosenoids.

Mass	Rt	Putative Metabolite	LPS/C		11E-OH + LPS/C		11E-ester + LPS/C		11E-acid + LPS/C	
			Ratio	*p*-Value	Ratio	*p*-Value	Ratio	*p*-value	Ratio	*p*-Value
		**Fatty Acid and Related Metabolites**								
270.220	15.12	3-oxo-hexadecanoic acid	1.018	ns	1.075	ns	1.154	0.003	1.141	0.006
244.167	6.55	Tridecanedioic acid	1.088	ns	1.268	ns	2.099	0.004	1.508	0.007
174.125	4.80	[FA hydroxy(9:0)] 2-hydroxy-nonanoic acid	1.241	ns	1.780	ns	2.082	0.017	1.813	0.012
342.277	21.13	Eicosanedioic acid*	0.979	ns	1.056	ns	1.118	0.034	1.167	0.010
396.397	29.61	hexacosanoic acid*	1.152	ns	1.070	ns	1.073	0.012	1.116	0.012
382.345	20.49	[FA hydroxy(24:0)] 2-hydroxy-15-tetracosenoic acid	0.981	ns	1.099	ns	1.176	0.001	1.148	0.013
244.204	11.64	2S-Hydroxytetradecanoic acid	0.740	<0.001	1.010	ns	0.920	ns	0.838	0.013
202.157	4.60	hydroxy-undecanoic acid	0.891	ns	4.096	<0.001	56.490	<0.001	2.130	0.032
270.256	20.57	heptadecanoic acid*	1.013	ns	1.043	ns	1.121	ns	1.087	0.044
156.079	25.56	5-oxo-7-octenoic acid	1.084	ns	1.156	ns	1.563	0.007	1.183	0.020
256.204	9.30	4-oxo-pentadecanoic acid	1.168	ns	1.262	ns	1.344	0.001	1.187	0.046
214.157	5.62	Oxododecanoic acid	1.072	ns	1.394	<0.001	1.465	<0.001	1.420	<0.001
354.313	20.75	10-oxo-docosanoic acid	1.013	ns	1.324	ns	8.329	<0.001	4.465	<0.001
346.235	12.85	9-hydroperoxy-12,13-dihydroxy-10-octadecenoic acid	0.935	ns	1.122	ns	100.996	0.010	1.858	ns
240.209	16.22	2,5-dimethyl-2E-tridecenoic acid	1.098	ns	1.029	ns	1.531	0.032	0.898	ns
340.298	17.05	2-oxo-heneicosanoic acid	1.007	ns	1.139	0.041	1.135	0.033	1.080	ns
426.371	26.35	Hexacosanedioic acid*	0.947	ns	1.035	ns	1.085	0.043	1.062	ns
216.172	8.55	Hydroxydodecanoic acid	0.564	0.001	0.946	ns	1.055	ns	0.904	ns
408.303	25.52	octacosaoctaenoic acid	2.604	0.029	1.653	ns	0.491	ns	1.717	ns
188.141	5.63	Hydroxydecanoic acid	0.747	0.040	1.334	ns	1.376	ns	1.173	ns
382.381	28.86	[FA (25:0)] pentacosanoic acid*	1.124	0.019	1.084	ns	1.106	0.011	1.128	0.001
389.260	9.17	N-(9Z-octadecenoyl)-taurine	0.924	ns	1.632	0.005	1.146	ns	1.143	ns
308.196	14.76	Trifluoro-11E-tetradecenyl acetate	1.599	0.007	1.524	0.027	2.133	0.004	1.484	0.017

300.266	14.33	9-methoxy-heptadecanoic acid	1.811	ns	1.328	ns	2.351	0.012	3.703	ns
268.204	7.94	3-oxo-2-pentyl-cyclopentanehexanoic acid	1.596	<0.001	1.155	ns	1.757	0.049	1.575	0.047
298.251	17.94	9-hydroxy-12Z-octadecenoic acid	1.059	ns	1.054	ns	1.080	0.011	1.100	0.037

Rt: Retention time (min); LPS: Lipopolysaccharides; *: Matches the analytical standard retention time; ns: Non-significant

Table S6: List of abbreviations used in this study.

List of Abbreviations	
HILIC	Hydrophilic Interaction Liquid Chromatography
RP	Reversed Phase
HPLC	High Performance Liquid Chromatography
LC-MS	liquid chromatography-mass spectrometry
ELISAs	Enzyme-linked immunosorbent assay
SIMCA	Soft-Independent Modelling of Class Analogy
OPLS-DA	Orthogonal Partial Least Squares Discriminant Analysis
PCA	Principal Component Analysis
QC	Quality control
RT	Retention Time
PLA2	Phospholipase A2
PBS	Phosphate Buffered Saline
KEGG	Kyoto Encyclopedia of Genes and Genomes
TCA	Cycle Tricarboxylic Acid cycle
OXPHOS	Oxidative phosphorylation
ATP	Adenosine Triphosphate
ADP	Adenosine Diphosphate
NAD+	Nicotinamide Adenine Dinucleotide (oxidised)
NADH	Nicotinamide Adenine Dinucleotide (reduced)
NADP+	Nicotinamide Adenine Dinucleotide phosphate (oxidised)
NADPH	Nicotinamide Adenine Dinucleotide phosphate (reduced)
F6P	Fructose-6-phosphate
G6P	Glucose-6-phosphate
G3P	glyceraldehyde-3-phosphate
S7P	Sedoheptulose 7-phosphate
IMP	Inosine monophosphate
AMP	Adenosine monophosphate
CDP	Cytidine diphosphate
CTP	Cytidine triphosphate
UTP	Uridine-5'-triphosphate
UDP	Uridine diphosphate
UMP	Uridine monophosphate
G6S	D-Glucose 6-sulfate
GLP	Glycerone phosphate
3PG	3-Phospho-D-glycerate
Arg. Succ.	N-(L-Arginino)succinate
PMA	Phorbol 12-myristate 13-acetate
PC	Phosphocholines
PI	Phosphoinositol
PS	Phosphoserines
PG	Phosphoglycerols
LPS	Lipopolysaccharide
PAMPs	Pathogen-associated molecular patterns
PRRs	Pattern Recognition Receptors
TLRs	Toll-like receptors
ROS	Reactive oxygen species
iNOS	Nitric oxide synthase
NO	Nitric oxide
BV	Bee venom
PGE2	Prostaglandin E2
Nuclear factor kappa B	NF-κB

Table S7: List of catalog/serial number of instruments and reagents used in this study.

Catalog/Serial Numbers	
HPLC	5035.0016
MS	SN01059P
Reveleris® iES system	1912L00078
plate reader	MV02120
ZIC-pHILIC column	543895
ACE C4 column	A73193
TNF- α ELISA Ready-Set-Go kits	88-7346-88
IL-1β ELISA Ready-Set-Go kits	88-7261-88
IL-6 ELISA Ready-Set-Go kits	88-7066-88
IL-10 ELISA Ready-Set-Go kits	88-7106-88
RPMI 1640 media	15-040-CVR
foetal calf serum	F13-1090/500
L-glutamine solution	RNBF8011
Penicillin/Streptomycin	015M4769V
